# Proposals to revise the Statutes of the International Committee on Systematics of Prokaryotes

**DOI:** 10.1099/ijsem.0.006291

**Published:** 2024-03-05

**Authors:** David R. Arahal, Markus Göker, Richard L. Hahnke, Célia M. Manaia, Edward R.B. Moore, Aharon Oren, Iain C. Sutcliffe, Stefano Ventura

**Affiliations:** 1 Departamento de Microbiología y Ecología, Universitat de València, 46100 Burjassot (Valencia), Spain; 2 Leibniz Institute DSMZ – German Collection of Microorganisms and Cell Cultures, Department of Bioinformatics and Databases, Inhoffenstrasse 7B, 38124 Braunschweig, Germany; 3 Leibniz Institute DSMZ – German Collection of Microorganisms and Cell Cultures, Department of Microorganisms, Inhoffenstrasse 7B, 38124 Braunschweig, Germany; 4 Universidade Católica Portuguesa, CBQF – Centro de Biotecnologia e Química Fina – Laboratório Associado, Escola Superior de Biotecnologia, Rua Diogo Botelho 1327, 4169-005 Porto, Portugal; 5 Department of Infectious Disease, and Culture Collection University of Gothenburg, Institute for Biomedicine, Sahlgrenska Academy, University of Gothenburg, P.O. Box 7193, SE0492 34, Gothenburg, Sweden; 6 Department of Plant and Environmental Sciences, The Institute of Life Sciences, The Hebrew University of Jerusalem, Edmond J. Safra Campus, Jerusalem 9190401, Israel; 7 Northumbria University, Newcastle Upon Tyne NE1 8ST, UK; 8 IRET-CNR, Research Institute on Terrestrial Ecosystems, National Research Council, Via Madonna del Piano 10, 50019 Sesto Fiorentino, and NBCF, National Biodiversity Future Center, Palermo, Italy

**Keywords:** ICSP, International Code of Nomenclature of Prokaryotes, statutes, taxonomy

## Abstract

The International Committee on Systematics of Prokaryotes serves to administer the rules of prokaryotic nomenclature via the International Code of Nomenclature of Prokaryotes, ensures the publication of the *International Journal of Systematic and Evolutionary Microbiology*, and works to represent the interests of the microbiological disciplines regarding prokaryotic nomenclature. The functions and mechanisms of operation of the International Committee on Systematics of Prokaryotes (ICSP) are defined in its Statutes, which were last revised in 2019. As members of the 2020–2023 and the 2023–2026 ICSP Executive Board and the Judicial Commission, we propose here some further revisions to help improve the clarity and functionality of the Statutes.

The International Committee on Systematics of Prokaryotes (ICSP) is a Committee of the Bacteriology and Applied Microbiology (BAM) Division of the International Union of Microbiological Societies (IUMS; https://iums.org/about-us/iums-structure.html). Its primary functions are to maintain the International Code of Nomenclature of Prokaryotes (ICNP) [1] as a ‘living document’, to represent the diverse interests of microbiological disciplines on matters concerning nomenclature, to oversee the publication of the *International Journal of Systematic and Evolutionary Microbiology* (IJSEM), to coordinate various Subcommittees on Taxonomy and its Judicial Commission, and to promote the discipline of microbial systematics. The functions of the ICSP, and how its subsidiaries must operate, are defined in its Statutes, which were last revised in 2019 [2]. These revisions have greatly improved the efficiency and effectiveness of the ICNP, not least through revision of the processes by which emendations to the ICNP are handled and by facilitating the increased use of electronic forums to conduct the activities of the ICSP.

Nevertheless, as members of the Executive Board of the ICSP (2020–2023, 2023–2026) and the Judicial Commission, we have noted that some further improvements to the Statutes are warranted. Article 17 of the ICSP Statutes states that ‘Five or more members of the ICSP may initiate amendment of the Statutes by submission of a manuscript to the IJSEM requesting amendment and providing the rationale’ [2]. Here, we propose amendments to the Statutes (Table S1, available in the online version of this article). Many are minor matters of grammatical or semantic clarification but others are more substantive. In the case of the latter, the rationale is given in Table S1. Of these, one proposal has already been considered by the members of the ICSP. In August 2022, the ICSP conducted a discussion of proposals to address a possible lack of diversity representation in the ICSP membership, including changes to the ICSP membership structure [3]. In January and February 2023, the voting members of the ICSP were balloted on a proposal that IUMS Member Societies would be permitted to delegate more than one member to ICSP, proportionate to their size. This membership model was approved in principle by the vote of the ICSP [4] and is here formally presented as a revision to Article 2(a)(3) (Table S1).

Other substantive changes proposed are as follows:

**Article 2(b)(1) and elsewhere:** it is clarified how the outcome of votes of the ICSP is to be determined.

**Article 2(d) and elsewhere:** some Statutes remain tied to the period when the ICSP Plenary was the principal vehicle for ICSP activities. Whilst we do not deny the importance of the ICSP Plenary, electronic forums are now the primary means by which the ICSP functions, so changes are proposed to reflect this.

**Article 4(d):** changes are proposed for the venues in which various minutes are published, reflecting the greater range of repositories that are available, including the ICSP website (https://www.the-icsp.org/).

**Article 6:** to reflect past practice, a formal process is proposed by which the ICSP can form *Ad Hoc* Subcommittees that focus on particular topics rather than specific taxa.

**Article 7(a) and elsewhere:** the mode of operation of the Judicial Commission is changed to allow full separation of powers between the legislature and the judiciary, thereby also simplifying the procedures.

**Article 8:** the criteria under which a Request for an Opinion is considered for publication in IJSEM are clarified. The processing of such a Request by the Judicial Commission is made simpler. The process by which appeals against decisions of the Judicial Commission can be made is clarified.

In accordance with the requirements of Article 17 of the ICSP Statutes, we present these proposals for further consideration and eventual ballot of the ICSP. The documents will be posted on the Slack channel at https://icnp-revision.slack.com for a 6-month discussion period starting on the day of publication of this announcement in the IJSEM.

## supplementary material

10.1099/ijsem.0.006291 Uncited Table S1.

## Abbreviations

BAM: bacteriology and applied microbiology division of IUMS
ICNP: International Code of Nomenclature of Prokaryotes
ICSP: International Committee on Systematics of Prokaryotes
IJSEM: *International Journal of Systematic and Evolutionary Microbiology*
IUMS: International Union of Microbiological Societies
