# Supplementary material for: Proposals to revise the Statutes of the International Committee on Systematics of Prokaryotes
Source: Int J Syst Evol Microbiol. 2024 Mar 5;74(3):006291. doi: 10.1099/ijsem.0.006291 (PMC10999737; doi:10.1099/ijsem.0.006291)
Supplement: Uncited Table S1. [file ijsem-74-06291-s001.pdf]

Supplementary Table 1: Proposals to revise the Statutes of the International Committee on Systematics of Prokaryotes

Differences between the current text and the proposed new text are highlighted yellow.

Current text	Proposed new text	Comments
**STATUTES OF THE INTERNATIONAL COMMITTEE ON SYSTEMATICS OF PROKARYOTES, ITS SUBCOMMITTEES AND THE JUDICIAL COMMISSION ON PROKARYOTE NOMENCLATURE OF THE BACTERIOLOGY AND APPLIED MICROBIOLOGY DIVISION OF THE IUMS**	**STATUTES OF THE INTERNATIONAL COMMITTEE ON SYSTEMATICS OF PROKARYOTES, ITS SUBCOMMITTEES AND THE JUDICIAL COMMISSION ON PROKARYOTE NOMENCLATURE OF THE BACTERIOLOGY AND APPLIED MICROBIOLOGY DIVISION OF THE IUMS**	
**Article 1**<br><br>**Name.** The name of the Committee shall be the International Committee on Systematics of Prokaryotes of the Bacteriology and Applied Microbiology (BAM) Division of the International Union of Microbiological Societies (IUMS). For the purpose of abbreviation in all official documents it will be referred to as the ICSP.	**Article 1**<br><br>**Name.** The name of the Committee shall be the International Committee on Systematics of Prokaryotes of the Bacteriology and Applied Microbiology (BAM) Division of the International Union of Microbiological Societies (IUMS). For the purpose of abbreviation in all official documents, it will be referred to as the ICSP.	
**Article 2**<br><br>Membership on the ICSP shall be **Full Members**, **Co-opted Members** and **Life Members**. Each Member Society (as defined in Article 3 of the constitution of the BAM Division of IUMS) of the BAM Division of the	**Article 2**<br><br>Membership on the ICSP shall be **Full Members**, **Co-opted Members** and **Life Members**. Each Member Society (as defined in Article 3 of the constitution of the BAM Division of IUMS) of the BAM Division of	

IUMS is entitled to appoint one Full Member to the Committee. The ICSP may co-opt other members and appoint Life Members.<br>(a) **Full Members**. Each Member Society of the BAM Division of the IUMS is entitled to appoint one Full Member to the ICSP by means of a letter to the Executive Secretary of the ICSP. Full Members may be appointed at any time. A Member Society is not obliged to appoint a member, and such positions will be considered to be vacant until a member is appointed.<br>(1) The tenure of a Full Member is for one three year term. A term begins upon appointment by a Member Society and ends either upon appointment of another Full Member by the Member Society or on 1 April 2020, or every three years thereafter. Full Members shall be eligible for reappointment by his or her Member Society for an indefinite period.<br>(2) When a Society ceases to be a member of the BAM Division of the IUMS, its appointed representative shall continue to be a Full Member until the end of his or her term.	the IUMS is entitled to appoint at least one Full Member to the Committee. The ICSP may co-opt other members and appoint Life Members.<br>(a) **Full Members.** Each Member Society of the BAM Division of the IUMS is entitled to appoint at least one Full Member to the ICSP by means of a letter to the Executive Secretary of the ICSP. Full Members may be appointed at any time. A Member Society is not obliged to appoint a member, and such positions will be considered to be vacant until a member is appointed.<br>(1) The tenure of a Full Member is for one three-year term. A term begins upon appointment by a Member Society and ends either upon appointment of another Full Member by the Member Society or on 1 April 2020, or every three years thereafter. Full Members shall be eligible for reappointment by their Member Society for an indefinite period.<br>(2) When a Society ceases to be a member of the BAM Division of the IUMS, its appointed representatives shall continue to be Full Members until the end of their term.<br>(3) The number of Full Members that can be appointed as delegates to the ICSP by a Member Society of the BAM Division of the IUMS is dependent on the number of members of the Member Society as follows: default, one delegate; 1-499 members, one delegate; 500-999 members, two	Addition reflects changes to Article 2(a)(3) below.<br><br>This amendment was approved in principle in ballot held in February 2023. A slightly different scaling compared to indicative range was cited on the ballot form. The change in scale is based on information regarding typical size of IUMS member societies.

	delegates; >1000 members, three delegates.<br>(4) For the purpose of appointing more than one delegate, a Member Society of the BAM Division of the IUMS must provide evidence for the current number of its own members to the Executive Secretary of the ICSP prior to the appointment.<br>(5) If the change in number of members of a Member Society affects the number of appointed delegates, all appointed delegates shall continue to be a Full Member until the end of their term.	
(b) **Co-opted Members.** The ICSP may co-opt members for the purpose of assisting with the work of the Committee.<br>(1) Proposals for co-opted membership must be submitted to the Executive Secretary of the ICSP and approved by a simple majority of the Full Members voting. Proposals should include a letter of nomination and two letters of support by Full Members.<br>(2) The term of a Co-opted Member shall be the same as Full Members.	(b) **Co-opted Members.** The ICSP may co-opt members for the purpose of assisting with the work of the Committee.<br>(1) Proposals for co-opted membership must be submitted to the Executive Secretary of the ICSP and approved by a relative majority (plurality) of the Full Members who vote. Proposals must include a letter of nomination giving the case for co-option. The letter of nomination can be issued by any party, including self-nominations. Any proposal must be supported in written form by at least two Full Members.<br>(2) The term of Co-opted Members shall be the same as for Full Members. If Co-opted Members wish to renew their membership, they should notify the Executive Secretary of the ICSP within four months of the start of a	This removes some ambiguity as to the type of majority needed. Similar edits made elsewhere for consistency.<br><br>This opens up the process slightly as a means to encourage participation in the ICSP.<br><br>This streamlines the process of renewal, which helps preserve diversity of membership.

(3) Co-opted Members have the right to vote in all matters except the election of officers and co-opted members.	new term, so that a ballot can be arranged, and provide a brief justification for renewal.<br>(3) Co-opted Members have the right to vote in all matters except the election of officers, Co-opted Members, and Life Members.	
(c) **Life Members.** The ICSP may appoint to Life Membership of the ICSP individuals who have rendered distinguished service to the ICSP. Such Life Members are not representatives of any Member Society. Life Members will not have a vote in the ICSP or be eligible for election to office and membership in the Executive Board.	(c) **Life Members.** The ICSP may appoint to Life Membership of the ICSP up to four individuals who have retired from their academic or scientific career and rendered distinguished service to the ICSP. Such Life Members are not representatives of any Member Society.<br>(1) Proposals for Life Membership must be submitted by ICSP Members to the Executive Secretary of the ICSP, with an appropriate justification and approved by a relative majority (plurality) of the Members eligible to vote.<br>(2) Life Members will have the right to vote in all ICSP matters except the election of officers, Co-opted Members and Life Members. Life Members will not be eligible for election to office and membership in the Executive Board.	Clarifies who can be appointed as a Life Member and sets an upper limit. Some of the authors proposed to limit the number of Life Members to maximal four, but no consensus was reached. We are looking forward to receiving comments on the Slack platform during the open discussion.<br><br>Specifies the actual mechanism by which Life Members can be proposed.<br><br>Gives Life Members voting rights similar to those of co-opted members.
(d) **Recognition of Alternates.** If a Member of the ICSP cannot attend meetings of the ICSP, an Alternate having all the rights of a Member, except that of eligibility to office in the ICSP, will be appointed in accordance with the following provisions.<br>(1) The Member Society that the member represents shall have the right to appoint an Alternate from within its own society or from within any other Member Society.	(d) **Recognition of Alternates.** If a Full Member of the ICSP cannot attend a meeting of the ICSP, an Alternate having all the rights of a Full Member, except that of eligibility to hold office in the ICSP, will be appointed in accordance with the following provisions.<br>(1) A Full Member of the ICSP who cannot attend the meeting shall have the right to appoint an Alternate from within the ICSP Member's own Member Society or, if this is not possible,	The necessity to appoint alternates is mostly a hangover from the era in which voting was conducted at the plenary meeting (every 3-4 years), whereas most voting is now by e-ballot ensuring delegate availability. Nevertheless, it may be desirable to ensure representation of a Member Society in a particular meeting and so this option is retained. The change simplifies the process by which alternates can be designated given that many/most Member Societies are

(2) If no appointment is made by the Member Society within two weeks of a request to appoint an Alternate, Full Members may appoint an Alternate from the Member Societies.<br>(3) Notice of appointment of Alternates shall be made to the Executive Secretary of the ICSP.	from within any other Member Society of the BAM Division of the IUMS.<br>(2) Notice of appointment of Alternates shall be made to the Executive Secretary of the ICSP prior to the meeting.	happy for their delegates to act autonomously. The amendment also clarifies that an alternate is appointed for a specific meeting and not permanently. The members of the EB should not be included in the issue of alternates. If the Chair is absent from a meeting, it is clear that the Vice-Chair would take over the duties.
(e) **Voting.** Full and Co-opted Members are entitled to only one vote of the Committee. Decisions require a vote of at least ten Members and shall be reached on the basis of a majority of the members voting, including abstentions. In ballots the decision shall be based on the votes received within the allotted time period and not on the number of members eligible to vote.	(e) **Voting.** Full and Co-opted Members are entitled to one vote of the Committee. Decisions require a vote of at least ten Members and shall be reached on the basis of a relative majority (plurality) of the members who voted. In ballots, the decision shall be based on the votes received within the allotted time period and not on the number of members eligible to vote. All ballots should have abstention as an option.	Clarifies how voting is conducted.
**Article 3**<br><br>**Functions of the ICSP**<br><br>(a) To represent the diversity of interests of different microbiological disciplines on matters concerning the nomenclature of prokaryotes.<br>(b) To write, edit, interpret and disseminate the *International Code of Nomenclature for Prokaryotes* (ICNP).<br>(1) To publish the ICNP.	**Article 3**<br><br>**Functions of the ICSP**<br><br>(a) To represent the diversity of interests of different microbiological disciplines on matters concerning the nomenclature of prokaryotes.<br>(b) To study, edit and disseminate the *International Code of Nomenclature for Prokaryotes* (ICNP). Interpretations of the Code that underlie the preparation and publication of Opinions are a task assigned by the ICSP to its Judicial Commission [see Article 8].<br>(1) To publish the ICNP [see Article 13].	Change proposed to implement separation of powers between legislature (voting members) and judiciary (JC) and to reduce bureaucracy.

(c) To establish publications deemed necessary for advancement of the systematics of prokaryotes.<br>(1) To maintain and disseminate the "Validation Lists" and the *Approved Lists of Bacterial Names*.<br>(2) To publish the *International Journal of Systematic and Evolutionary Microbiology* (IJSEM).	(c) To establish publications deemed necessary for advancement of the systematics of prokaryotes.<br>(1) To maintain and disseminate the "Validation Lists" and the *Approved Lists of Bacterial Names* [see Article 9].<br>(2) To oversee publication of the *International Journal of Systematic and Evolutionary Microbiology* (IJSEM) [see Article 14].	Additions proposed here intend to better reflect the tasks of the ICSP and to clarify connections to other articles of the Statutes.
(d) To elect members of the Judicial Commission as vacancies occur and to replace the members of the several classes as their terms expire.	(d) To elect members of the Judicial Commission as vacancies occur and to replace the members of the several classes as their terms expire.	
(e) To consider and vote upon all recommendations made by the Judicial Commission relating to Requests for Opinions. To consider and vote upon all recommendations communicated by the Editor-in-Chief of the ICNP for emendation of the ICNP.	(e) To consider and vote upon all recommendations made by the Judicial Commission relating to Requests for Opinions. To consider and vote upon all recommendations communicated by the Editor-in-Chief of the ICNP for emendation of the ICNP [see Article 13].	
(f) To receive reports of the Subcommittees on Taxonomy and ensure their dissemination.	(f) To receive reports of the Subcommittees on Taxonomy and ensure their dissemination [see Article 6].	Change proposed to implement separation of powers between legislature (voting members) and judiciary (JC) and to reduce bureaucracy.
(g) To organize and sponsor regional, national or international conferences on the systematics of prokaryotes, either independently or in conjunction with meetings organized by other professional societies. Conferences may be held electronically and made available via the Internet or other means as technology advances.	(g) To organize and sponsor regional, national or international conferences on the systematics of prokaryotes, either independently or in conjunction with meetings organized by other professional societies. Conferences may be held electronically and made available via the Internet or other means as technology advances [see Article 4].	
(h) To elect the Executive Board (EB) of the ICSP and to authorize the EB-ICSP to perform such	(h) To elect the Executive Board (EB) of the ICSP and to authorize the EB-ICSP to perform such functions as are defined in the Statutes and	

functions as are defined in the Statutes and such other functions as the ICSP may determine.<br><br>(i) To appoint as Life Members of the ICSP individuals who have rendered distinguished service to the ICSP.<br>(j) To take other actions that ensure the proper application of the Code.	such other functions as the ICSP may determine [see Article 5].<br>(i) To vote on nominations of Co-opted Members of the ICSP [see Article 2(b)].<br>(j) To appoint as Life Members of the ICSP individuals who have rendered distinguished service to the ICSP [see Article 2(c)].<br>(k) To take other actions that ensure the proper application of the Code.	
**Article 4**<br><br>**Conduct of Business by the ICSP**<br><br>(a) The business of the ICSP will be conducted at Plenary Meetings or electronic forums. Plenary Meetings will be held at such times and places as may be determined by the EB-ICSP. When possible, Plenary Meetings shall be held at intervals of not more than three years and shall be held in association with other meetings, conferences, or congresses sponsored by the ICSP, IUMS, or other scientific societies.<br>(b) All committees, subcommittees, and commissions of the ICSP have the authority to conduct business by electronic media.<br>(c) When conducting the business of the ICSP in electronic media, the format of the meeting is chosen by the Executive Board. Generally, time should be allowed for sharing the relevant materials on the topic under discussion, deliberations by the members, and voting.	**Article 4**<br><br>**Conduct of Business by the ICSP**<br><br>(a) The business of the ICSP will be conducted at Plenary Meetings or electronic forums. Plenary Meetings will be held at such times and places as may be determined by the EB-ICSP. When possible, Plenary Meetings shall be held at intervals of not more than three years and shall be held in association with other meetings, conferences, or congresses sponsored by the ICSP, IUMS, or other scientific societies.<br>(b) All committees, subcommittees, and commissions of the ICSP have the authority to conduct business by electronic media.<br>(c) When conducting the business of the ICSP in electronic media, the format of the meeting is chosen by the Executive Board. Generally, time should be allowed for sharing the relevant materials on the topic under discussion, deliberations by the members, and voting.	

(d) The business of the ICSP will be conducted publicly, with minutes reported of all meetings of the ICSP and its committees. Minutes of the Plenary Meeting, Judicial Commission, Subcommittees on Taxonomy will be reported by publication in the IJSEM. Minutes of the EB-ICSP, editorial boards and other committees may be made publicly available by other means, including posting on a publicly accessible website or listserv, as determined by the committees.	(d) The business of the ICSP will be conducted publicly, with minutes reported of all meetings of the ICSP and its committees. Minutes of the Plenary Meeting will be reported by publication in the IJSEM. Minutes of the EB-ICSP, Judicial Commission, Subcommittees, editorial boards and other committees will be made publicly available by appropriate means, possibly including posting on a permanently publicly accessible website or repository, as determined by the committees.	The plenary is the main meeting of the ICSP and so those minutes should be published in IJSEM. However, it seems sufficient that routine minutes of the subsidiary components should be circulated by other means including email distribution, our own website and the 'ICSP community' within Zenodo (which also provides documents with a DOI). Opinions of the JC are considered separately below.
**Article 5**<br><br>**The Executive Board-ICSP.** The membership of the Executive Board (EB) shall be: Chair and Vice-Chair, Executive Secretary, Secretary for Subcommittees on Taxonomy, Treasurer, and two other Full Members. The Chair, Vice-Chair, and Secretary of the Judicial Commission shall be ex officio non-voting members of the Executive Board. No single member may hold more than one function or cast more than one vote. No member of the EB-ICSP may serve on the Executive Board for more than two consecutive terms in any capacity.<br><br>(a) **Election of the Executive Board.** The terms of office for members of the EB-ICSP will begin on September 1, 2020, and every three years	**Article 5**<br><br>**The Executive Board-ICSP.** The membership of the Executive Board (EB) shall be: Chair and Vice-Chair, Executive Secretary, Secretary for Subcommittees on Taxonomy, Treasurer, and two other Full Members. The Chair, Vice-Chair, and Secretary of the Judicial Commission shall be ex officio non-voting members of the Executive Board. No single member may hold more than one function or cast more than one vote. No member of the EB-ICSP may serve on the Executive Board for more than two consecutive terms; after an absence of one term, former members of the EB-ICSP who have served two terms on the Executive Board shall be eligible again for election as an Officer.<br><br>a) **Election of the Executive Board.** The terms of office for members of the EB-ICSP begin on September 1, 2020, and every three years	It was agreed to propose this clarification at the April 2023 meeting of the Executive Board.

thereafter. One term is for three years. Members of the EB who are Full Members or Co-opted Members at the beginning of their term will retain their membership status until the end of their term even if their Member Society appoints another Member. The Chair and Vice-Chair will be elected from the Full Members. The Executive Secretary, the Secretary for Subcommittees on Taxonomy, and the Treasurer will be elected from the Full or Coopted Members.	thereafter. One term is for three years. Members of the EB who are Full Members or Co-opted Members at the beginning of their term will retain their membership status until the end of their term, even if their Member Society appoints another Member. The Chair and Vice-Chair will be elected from the Full Members. The Executive Secretary, the Secretary for Subcommittees on Taxonomy, and the Treasurer will be elected from the Full or Co-opted Members.	
(b) **Functions of the EB-ICSP**<br>(1) To organize meetings and electronic forums of the ICSP plenary and prepare and publish agenda for such meetings and forums.<br>(2) To conduct the business of the ICSP between plenary meetings as directed by the ICSP.<br>(3) To organize regional, national and international conferences and symposia on the systematics of prokaryotes.<br>(4) To appoint Subcommittees on Taxonomy, either on its own initiative or at the request of others, and to facilitate meetings of the Subcommittees on Taxonomy at the plenary meetings of the ICSP or elsewhere.<br>(5) To provide for the initial appointment and subsequent election of Chairs and Secretaries of Subcommittees on Taxonomy.<br>(6) To deal with such business as the Judicial Commission may from time to time request.	(b) **Functions of the EB-ICSP**<br>(1) To organize meetings and electronic forums of the ICSP plenary [see Article 4(a)].<br>(2) To conduct the business of the ICSP between plenary meetings as directed by the ICSP [see Article 4(b)].<br>(3) To organize regional, national and international conferences and symposia on the systematics of prokaryotes or other appropriate topics.<br>(4) To appoint Subcommittees [see Article 6], either on its own initiative or at the request of others, and to facilitate meetings of the Subcommittees at the plenary meetings of the ICSP or elsewhere.<br>(5) To provide for the initial appointment and subsequent election of Chairs and Secretaries of Subcommittees on Taxonomy [see Article 6].<br>(6) To deal with such business as the Judicial Commission may request [see Article 7].	Additions proposed here intend to better reflect the tasks of the EB-ICSP and to clarify connections to other articles of the Statutes.<br><br>Change in agreement with the one proposed below, using “Subcommittee” to collectively refer to Subcommittees on Taxonomy and Ad Hoc Subcommittees.

(7) To recommend to the ICSP the establishment of any Committees that are deemed necessary for the work and functioning of the ICSP and to consider recommendations submitted to it by those committees.	(7) To recommend to the ICSP the establishment of any Committees that are deemed necessary for the work and functioning of the ICSP and to consider recommendations submitted to it by those committees.	
	(8) To appoint or replace individuals to implement and keep up to date the representation of the ICSP on electronic media, such as websites or social media, as deemed appropriate for the purposes of the ICSP.	Change intended to clarify that the ICSP should be active in electronic media.
(8) Upon approval of the ICSP to:<br>(a) establish publications authorized by the ICSP<br>(b) negotiate such contracts as may be necessary for the issuance of publications authorized by the ICSP<br>(c) establish such Trusts or enter into such agreements as may be advisable for the auditing and administration of funds that may be designated for the payment of the necessary operating expenses of the ICSP and its subordinate agencies, whether such funds originate from grants, gifts, royalties, the sale of publications or other sources.<br>(d) To request from appropriate agencies grants for the necessary expenses of the ICSP and its subordinate agencies.<br>(9) To ensure the proper functioning of the organizations and officers of the ICSP.<br>(10) To perform such other functions enumerated in the Statutes or assigned to it by the ICSP.	(9) Upon approval of the ICSP to:<br>(a) establish publications authorized by the ICSP [see Articles 9 and 14].<br>(b) negotiate such contracts as may be necessary for the issuance of publications authorized by the ICSP.<br>(c) establish such Trusts or enter into such agreements as may be advisable for the auditing and administration of funds that may be designated for the payment of the necessary operating expenses of the ICSP and its subordinate agencies, whether such funds originate from grants, gifts, royalties, the sale of publications or other sources.<br>(d) request from appropriate agencies grants for the necessary expenses of the ICSP and its subordinate agencies.<br>(10) To ensure the proper functioning of the organizations and officers of the ICSP.<br>(11) To perform such other functions enumerated in the Statutes or assigned to it by the ICSP.	

(c) **Duties of the Chair and Vice-Chair of the ICSP.** In addition to the duties specified below, the Chair and Vice-Chair shall be ex officio, but non-voting, members of all Committees, Commissions and Subcommittees of the ICSP, unless otherwise stated.<br>(1) Duties of the Chair of the ICSP are:<br>(a) To preside at meetings of the ICSP and its Executive Board.<br>(b) With the other members of the Executive Board, to prepare the agenda for meetings of the EB-ICSP.<br>(c) With the other members of the Executive Board, to prepare the agenda for meetings and forums of the ICSP.<br>(d) To serve on the Publications Committee.<br>(e) To serve on the Editorial Board for the ICNP.<br>(f) To assume such duties as may be requested by the ICSP or determined by the functions of the EB-ICSP.<br>(2) Duties of the Vice-Chair are:<br>(a) To preside at the meetings of the ICSP and EB-ICSP in the absence of the Chair.<br>(b) With the other members of the Executive Board to prepare the agenda for the meetings and forums of the ICSP.	(c) **Duties of the Chair and Vice-Chair of the ICSP.** In addition to the duties specified below, the Chair and Vice-Chair shall be ex officio, but non-voting, members of all Committees, Commissions and Subcommittees of the ICSP, unless otherwise stated.<br>(1) Duties of the Chair of the ICSP are:<br>(a) To preside at meetings of the ICSP and its Executive Board [see Article 4].<br>(b) With the other members of the Executive Board, to prepare the agenda for meetings of the EB-ICSP [see Article 4(d)].<br>(c) With the other members of the Executive Board, to prepare the agenda for meetings and forums of the ICSP [see Article 4(a)].<br>(d) To serve on the Publications Committee [see Article 9].<br>(e) To serve on the Editorial Board for the ICNP [see Article 13].<br>(f) To assume such duties as may be requested by the ICSP or determined by the functions of the EB-ICSP.<br>(2) Duties of the Vice-Chair are:<br>(a) To preside at the meetings of the ICSP and EB-ICSP in the absence of the Chair [see Article 4(d)].<br>(b) With the other members of the Executive Board to prepare the agenda for the meetings and forums of the ICSP [see Article 4(a)].	Additions proposed here intend to clarify connections to other articles of the Statutes.

(c) To assume such duties as may be requested by the ICSP or determined by the functions of the EB-ICSP.<br>(d) To chair the Publications Committee.<br>(e) To serve on the Editorial Board for the ICNP and IJSEM.	(c) To assume such duties as may be requested by the ICSP or determined by the functions of the EB-ICSP.<br>(d) To chair the Publications Committee [see Article 9].<br>(e) To serve on the Editorial Board for the ICNP and IJSEM [see Articles 13 and 14].	
(d) **Duties of the Executive Secretary.** In addition to the duties specified below, the Executive Secretary shall be responsible to the Executive Board in all matters associated with communications between the ICSP and the Executive Board of the IUMS, Division Council of BAM and Member Societies. The Executive Secretary shall perform the following duties:<br>(1) Be Secretary of the EB-ICSP and the ICSP and submit the minutes of the plenary meetings for publication in the IJSEM.<br>(2) Be secretary to the electronic meetings of the EB-ICSP and ICSP and report the minutes in an appropriate manner as determined by the plenary of the ICSP or, in the absence of specific instructions, by the EB-ICSP.<br>(3) With the other members of the EB-ICSP, to prepare the agenda for meetings of the EBICSP.<br>(4) Prepare, in cooperation with other members of the EB-ICSP, the agenda for meetings and forums of the ICSP.	(d) **Duties of the Executive Secretary.** In addition to the duties specified below, the Executive Secretary shall be responsible to the Executive Board in all matters associated with communications between the ICSP and the Executive Board of the IUMS, Division Council of BAM and Member Societies. The Executive Secretary shall perform the following duties:<br>(1) Be Secretary of the EB-ICSP and the ICSP and submit the minutes of the plenary meetings for publication in the IJSEM [see Article 4(a)].<br>(2) Be secretary to the electronic meetings of the EB-ICSP and ICSP and report the minutes in an appropriate manner as determined by the plenary of the ICSP or, in the absence of specific instructions, by the EB-ICSP [see Article 4(d)].<br>(3) With the other members of the EB-ICSP, to prepare the agenda for meetings of the EB-ICSP [see Article 4(d)].<br>(4) Prepare, in cooperation with other members of the EB-ICSP, the agenda for meetings and forums of the ICSP [see Article 4(a)].	Additions proposed here intend to clarify connections to other articles of the Statutes.

(5) Request nominations from Member Societies for Full Members to the ICSP. Receive nominations for appointment of Full, Co-opted and Life Members and transmit these to the EBICSP.<br>(6) Receive nominations for alternates for meetings of the ICSP in accordance with Article 2d.<br>(7) Transmit to the ICSP such recommendations from the Judicial Commission as may require action by the ICSP.<br>(8) If the members are asked to vote upon any proposal, to tabulate and announce the result of the ballot and to certify the result to the Chair of the ICSP and to the Chair of the Judicial Commission.<br>(9) Prepare every three years a complete list of all members and officers of the ICSP and the Judicial Commission and forward these to the Secretary Treasurer of the BAM Division of IUMS and the Chair of the ICSP.<br>(10) Present every three years a report covering all pertinent actions of the ICSP and the Judicial Commission and other committees of the ICSP to the plenary of the ICSP and the Secretary of the BAM Division of the IUMS.<br>(11) To maintain an archive of the important documents of the ICSP, including names and affiliations of its members and officers, minutes of meetings, copies of contracts,	(5) Request nominations from Member Societies for Full Members to the ICSP. Receive nominations for appointment of Full, Co-opted and Life Members and transmit these to the EB-ICSP.<br>(6) Receive nominations for alternates for meetings of the ICSP in accordance with Article 2d.<br>(7) Transmit to the ICSP such recommendations from the Judicial Commission as may require action by the ICSP [see Article 7(a)].<br>(8) If the members are asked to vote upon any proposal, to tabulate and announce the result of the ballot and to certify the result to the Chair of the ICSP and to the Chair of the Judicial Commission.<br>(9) Prepare every three years a complete list of all members and officers of the ICSP and the Judicial Commission and forward these to the Secretary Treasurer of the BAM Division of IUMS and the Chair of the ICSP.<br>(10) Present every three years a report covering all pertinent actions of the ICSP and the Judicial Commission and other committees of the ICSP to the plenary of the ICSP and the Secretary of the BAM Division of the IUMS.<br>(11) To maintain an archive of the important documents of the ICSP, including names and affiliations of its members and officers, minutes of meetings, copies of contracts,	

correspondence regarding any prizes awarded by the ICSP, reports of the treasurer, and documents related to ad hoc committees.<br>(12) Perform such duties as the EB-ICSP may from time to time determine.	correspondence regarding any prizes awarded by the ICSP, reports of the treasurer, and documents related to Ad Hoc Subcommittees.<br>(12) Perform such duties as the EB-ICSP may from time to time determine.	Change in agreement with the one proposed below, using the term "Subcommittee" to collectively refer to Subcommittees on Taxonomy and Ad Hoc Subcommittees.
(e) **Duties of the Treasurer.** The Treasurer shall receive funds which may be made available to the ICSP from any source and distribute them as directed by the EBICSP or its Chair.<br>(1) Checks or requests for electronic fund transfers issued by the Treasurer shall bear the Treasurer's signature but must have been authorized in writing by the Chair of the ICSP, the Vice-Chair of the ICSP or the Chair of the Judicial Commission.<br>(2) A statement of accounts shall be furnished by the Treasurer to the EB-ICSP before September 1 of each year. In addition, a statement will be furnished to the ICSP at each Plenary Meeting of the ICSP and to the Secretary Treasurer of the BAM Division of the IUMS as requested.	(e) **Duties of the Treasurer.** The Treasurer shall receive funds which may be made available to the ICSP from any source and distribute them as directed by the EB-ICSP or its Chair.<br>(1) Checks or requests for electronic fund transfers issued by the Treasurer shall bear the Treasurer's signature but must have been authorized in writing by the Chair of the ICSP, the Vice-Chair of the ICSP or the Chair of the Judicial Commission.<br>(2) A statement of accounts shall be furnished by the Treasurer to the EB-ICSP before September 1 of each year. In addition, a statement will be furnished to the ICSP at each Plenary Meeting of the ICSP and to the Secretary Treasurer of the BAM Division of the IUMS as requested.	
(f) **Duties of the Secretary for Subcommittees on Taxonomy.** The Secretary for Subcommittees on Taxonomy hall be responsible to the Executive Board for all matters associated with the Subcommittees on Taxonomy of the ICSP. The Secretary for Subcommittees on Taxonomy shall perform the following duties:	(f) **Duties of the Secretary for Subcommittees on Taxonomy.** The Secretary for Subcommittees on Taxonomy shall be responsible to the Executive Board for all matters associated with the Subcommittees of the ICSP. The Secretary for Subcommittees on Taxonomy shall perform the following duties:	Adaptation proposed in agreement with the new Article 6. The title of the office itself has been kept.

(1) Prepare, in cooperation with the Chair of the ICSP and the Executive Secretary, the agenda for meetings of the EB-ICSP.<br>(2) Prepare, in cooperation with other members of the EB-ICSP, the agenda for meetings and forums of the ICSP.<br>(3) Serve as a non-voting member of each Subcommittee on Taxonomy.<br>(4) Act as a liaison between the officers of the Subcommittees on Taxonomy and the ICSP, transmitting requests and recommendations to the EB-ICSP or Judicial Commission, whichever is appropriate.	(1) Serve as a non-voting member of each Subcommittee [see Article 6].<br>(2) Act as a liaison between the officers of the Subcommittees and the ICSP, transmitting requests and recommendations to the EB-ICSP and/or Judicial Commission, whichever is appropriate<br>(3) Organize, where needed, meetings of the officers of the Subcommittees using electronic forums, to discuss overarching matters.	These are the duties of the Chair and Vice Chair in conjunction "with the other members of the Executive Board", as described above (article 5.c.1). Hence can be deleted here for simplicity.<br><br>Change in agreement with the one proposed below, using the term "Subcommittee" to collectively refer to Subcommittees on Taxonomy and Ad Hoc Subcommittees.<br><br>A recent meeting of this type was held in October 2022 and considered useful; hence, this activity is formalized here.
**Article 6**<br><br>**Organization and Functions of Subcommittees on Taxonomy**<br><br>The formation of a Subcommittee on Taxonomy may be proposed by an individual, a group of individuals or the EBICSP. A proposal must be accompanied by a description of the taxa to be studied, the reasons therefore, and a list of proposed subcommittee members. Such proposals shall be submitted to the Secretary for Subcommittees on Taxonomy for approval by the EB-ICSP. Subcommittees on Taxonomy shall work under the following rules:	**Article 6**<br><br>**Organization and Functions of Subcommittees on Taxonomy and Ad Hoc Subcommittees**<br><br>The formation of a Subcommittee on Taxonomy or an Ad Hoc Subcommittee may be proposed by an individual, a group of individuals or the EB-ICSP. A proposal must be accompanied by a description of the prokaryotic taxa or, alternatively, the overarching nomenclature-related topic to be studied, the reasons therefore, and a list of proposed Subcommittee members. Such proposals shall be submitted to the Secretary for Subcommittees for approval by the EB-ICSP.	The ICSP has used ad hoc committees (and working groups) in the past. The additions here serve to formalize the status of these.<br><br>We have suggested here that ad hoc committees be called "Ad Hoc Subcommittees" to emphasize their position within the ICSP. The term "Subcommittee" can then be used to refer collectively to the Taxonomy Subcommittee and the Ad Hoc Subcommittees.

(a) Upon formation of a Subcommittee on Taxonomy, the members will elect a Chair and Secretary and report their names to the Secretary for Subcommittees on Taxonomy. The terms of the officers of the subcommittees begin on 1 September 2020, and every three years thereafter. However, they may be re-elected, and there is no term limit. The functions of the Chair are to organize with the Secretary meetings of the Subcommittee, chair such meetings, and coordinate all business as necessary with the ICSP and the Judicial Commission. The functions of the Secretary are to assist the Chair in organizing the subcommittee meetings, prepare minutes of the meetings for publication in the IJSEM, and maintain a list of the members of the Subcommittee.	Subcommittees shall work under the following rules:<br>(a) Upon formation of a Subcommittee, the members will elect a Chair and Secretary and report their names to the Secretary for Subcommittees on Taxonomy. The terms of the officers of the subcommittees begin on 1 September 2020, and every three years thereafter. However, they may be re-elected, and there are no term limits. The functions of the Chair are to organize, with the Secretary, meetings of the Subcommittee, chair such meetings, and coordinate all business as necessary with the ICSP and the Judicial Commission. The functions of the Secretary are to assist the Chair in organizing the Subcommittee meetings, prepare minutes of the meetings for publication [see Article 4(d)] and maintain a list of the members of the Subcommittee.	
(b) Regular members of a Subcommittee on Taxonomy shall be affiliated with an IUMS member society. The subcommittee may co-opt other specialists, but co-opted members have no voting rights in the administrative workings of the subcommittee. New members may be elected to a Subcommittee on Taxonomy at any time by a vote of the existing regular members.	(b) Regular members of a Subcommittee shall be affiliated with an IUMS member society. The Subcommittee may co-opt other specialists. New members may be elected to a Subcommittee at any time by a vote of the existing regular members.	Simplification intended to give the Subcommittees more flexibility.
(1) The Secretary for Subcommittees on Taxonomy shall be a non-voting member of each Subcommittee on Taxonomy and acts as liaison between each Subcommittee on Tax	(1) The Secretary for Subcommittees on Taxonomy shall be a non-voting member of each Subcommittee and acts as liaison	

nomy, the EB-ICSP, the ICSP and the Judicial Commission.<br>(2) Members who cannot attend meetings of a Subcommittee on Taxonomy may designate an Alternate in a letter to the Subcommittee's secretary. The designated Alternate may vote on the member's behalf. However, no member or alternate of the subcommittee may cast more than one vote.<br>(3) There is no term limit on membership in a Subcommittee on Taxonomy, but all members should be actively engaged in the work of the subcommittee. Members who resign or who have ceased to interest themselves in the work of the subcommittee shall be removed from the membership roster by a majority vote of the Subcommittee.<br>(4) Each Subcommittee on Taxonomy is encouraged to meet at least once every three years at any location considered appropriate by the subcommittee's members or hold electronic forums. Meetings and forums should be coordinated with the Secretary for Subcommittees on Taxonomy. A portion of the meeting may be restricted to regular members of the subcommittee or their alternates in order to deal, inter alia, with changes of membership and election of officers.	between each Subcommittee, the EB-ICSP, the ICSP and the Judicial Commission.<br>(2) Members who cannot attend meetings of a Subcommittee may designate an Alternate in a letter to the Subcommittee's secretary. The designated Alternate may vote on the member's behalf. However, no member or alternate of the subcommittee may cast more than one vote.<br>(3) There is no term limit on membership in a Subcommittee, but all members should be actively engaged in the work of the Subcommittee. Members who have ceased to interest themselves in the work of the Subcommittee shall resign or be removed from the membership roster by a relative majority (plurality) vote of the Subcommittee.<br>(4) Each Subcommittee is encouraged to meet at least once every year at any location considered appropriate by the Subcommittee's members or hold electronic forums. Meetings and forums should be coordinated with the Secretary for Subcommittees on Taxonomy. A portion of the meeting may be restricted to regular members of the subcommittee or their alternates in order to deal, inter alia, with changes of membership and election of officers. Minutes of meetings will be communicated to the Secretary for	Online meetings are now routine and easily organized so it seems appropriate to encourage meetings more frequently.<br><br>Clarification on the handling of minutes in line with the proposed amendment to Article 4(d).

(5) Subcommittees on Taxonomy shall work within the framework provided by the International Code of Nomenclature of Prokaryotes (ICNP) in order to:<br>(a) Encourage and undertake research on the evolutionary relationships among the organisms in the taxa under study.<br>(b) Evaluate and make recommendations regarding characters or procedures useful in distinguishing the organisms and taxa under study.<br>(c) Make recommendations regarding classification of the organisms in the taxa under study. A Subcommittee on Taxonomy cannot legislate on classification but may contribute materially towards the general acceptance of a classification.<br>(d) Make recommendations to the JC regarding nomenclature for the organisms in the taxa under study, including recommendations for the conservation or rejection of names.<br>(e) Offer advice on recognition of the nomenclatural types of various taxa.<br>(f) Make recommendations regarding emendations of the ICNP.<br>(g) Recommend minimal standards for the description of new taxa. Such recommendations shall include a list of	Subcommittees on Taxonomy and published using appropriate means [see Article 4(d)].<br>(5) Subcommittees on Taxonomy, which focus on particular taxa, shall work within the framework provided by the ICNP to:<br>(a) Encourage and undertake research on the evolutionary relationships among the organisms in the taxa under study.<br>(b) Evaluate and make recommendations regarding characters or procedures useful in distinguishing the organisms and taxa under study.<br>(c) Make recommendations regarding classification of the organisms in the taxa under study. A Subcommittee on Taxonomy cannot legislate on classification but may contribute materially towards the general acceptance of a classification.<br>(d) Make recommendations to the JC regarding nomenclature for the organisms in the taxa under study, including recommendations for the conservation or rejection of names.<br>(e) Offer advice on recognition of the nomenclatural types of various taxa.<br>(f) Recommend minimal standards for the description of new taxa. Such recommendations shall include a list of	Clarification of the difference to Ad Hoc Subcommittees.<br>Any interested party can do this as per Article 13.2 so it need not be designated as a specific function of the Subcommittees.

characters and procedures for their assessment and shall be reviewed at regular intervals. The standards shall be published in the IJSEM and/or other microbiological journals. They shall be minimal and in no way limit the extent of investigation or contravene Principle 1 (4) of the ICNP.<br>(h) Maintain a list of validly published and other names that have been applied to the organisms in the taxa under study.<br>(i) While statements relating to such studies and recommendations may appear in subcommittee minutes, they may also be submitted separately for publication in the IJSEM.	characters and procedures for their assessment and shall be reviewed at regular intervals. The standards shall be published in the IJSEM and/or other microbiological journals. Such standards shall be minimal and in no way limit the extent of investigation or contravene Principle 1(4) of the ICNP.<br>(g) Maintain a list of validly published and other names that have been applied to the organisms in the taxa under study.<br>(h) Include statements and recommendations relating to the above studies in Subcommittee minutes or submit them separately for publication in the IJSEM or elsewhere, as appropriate [Article 4(d)].<br>(6) Ad Hoc Subcommittees, which focus on an overarching nomenclature-related topic, shall work within the framework provided by the ICNP to:<br>(a) Evaluate and make recommendations regarding the overarching nomenclature-related topic which is their remit, as approved by the Executive Board.<br>(b) Cooperate with other committees or similar bodies appointed to consider problems related to the overarching nomenclature-related topic.<br>(c) Make recommendations regarding emendations of the ICNP.	This is where the new clauses begin to formalize the role of Ad Hoc Subcommittees.

	(d) Publish recommendations for addressing the overarching nomenclature-related topic in the IJSEM and/or other microbiological journals.	In contrast to the comment above (regarding deleted article 5.f), this is included here as Ad Hoc Subommittees may be formed to do exactly this.
(c) **Dissolution of a Subcommittee on Taxonomy.**<br><br>(1) Subcommittees on Taxonomy may be dissolved if the members no longer consider that their work is required. The Secretary for Subcommittees on Taxonomy shall be informed.<br><br>(2) Subcommittees on Taxonomy that have not produced minutes over a five-year period will be considered inactive and dissolved by action of the EB-ICSP if no Chair, Secretary or members can be identified to continue the work.	(c) **Dissolution of a Subcommittee on Taxonomy or an Ad Hoc Subcommittee.**<br>(1) Subcommittees on Taxonomy and Ad Hoc Subcommittees may dissolve themselves if the members no longer consider that their work is required. The Secretary for Subcommittees on Taxonomy shall be informed.<br>(2) Subcommittees that have not produced minutes over a five-year period will be considered inactive and dissolved by action of the EB-ICSP if no Chair, Secretary or members can be identified to continue the work.<br><br>(3) Ad Hoc Subcommittees will report regularly to the EB-ICSP via the Secretary for Subcommittees on Taxonomy and if appropriate using other means [see Article 4(d)] until completion of their remit, at which point a summary report will be prepared for submission to the ICSP and, if appropriate, publication in IJSEM or on other suitable platforms [see Article 4(d)].	Additions in line with the formalisation of the role of Ad Hoc Subcommittees.
**Article 7**<br><br>**The Judicial Commission**	**Article 7**<br><br>**The Judicial Commission**	

(a) **Functions of the Judicial Commission.** The Judicial Commission (JC) is a subsidiary of the ICSP and has the following functions:	(a) **Functions of the Judicial Commission.** The Judicial Commission (JC) is the judiciary of the ICSP and has the following functions:	
(1) To provide the ICSP expert advice on the interpretation of the ICNP, Requests for Opinions, and other matters requested of it.	(1) To provide the ICSP expert advice on the interpretation of the ICNP, Requests for Opinions, and other matters requested of it. To publish guidelines on the interpretation of the ICNP as deemed necessary.	This formalizes the recent publication of the first Judicial Commission Guidelines; https://doi.org/10.1099/ijsem.0.005782
(2) To consider all Requests for Opinions relative to the interpretation of the ICNP. An Opinion shall be issued when seven or more Commissioners vote in favour of issuance. All Opinions shall be submitted to the ICSP for approval.	(2) To consider all Requests for Opinions relative to the interpretation of the ICNP [see Article 8]. An action of the JC, such as the rejection or conservation of a name, shall only be issued only when a majority of the Commissioners vote in favour. A tied vote is resolved by the vote of the three officers of the JC.	Changes are proposed to implement separation of powers between legislature (voting members) and judiciary (JC) and to reduce bureaucracy. Further adaptations regarding the processing of Requests for an Opinion depend on that. Change proposed to clarify how to proceed in the rare event of a tied vote.
(3) To consider proposals for emendation of the ICNP and make recommendations to the ICSP.	(3) To consider proposals for emendation of the ICNP and make recommendations to the ICSP.	
(4) To hold such sessions and electronic meetings as may be necessary to transact business.	(4) To hold such sessions and electronic meetings as may be necessary to transact business.	
(5) To report to the EB-ICSP the names of all Commissioners whose terms of service will expire and a list of other vacancies in the membership of the Commission.	(5) To report to the EB-ICSP the names of all Commissioners whose terms of service will expire or any other vacancies in the membership of the Commission (e.g., due to resignation).	Clarifies when vacancies may occur.
(6) To cooperate with other Commissions or similar bodies appointed to consider problems of nomenclature.	(6) To cooperate with other commissions or similar bodies appointed to consider problems of nomenclature.	
(7) To consider recommendations from Subcommittees on Taxonomy or other	(7) To consider enquiries from the wider scientific community on matters related to	Broadens out the constituency from which enquiries may be received.

specialists for the acceptance of lists of names as validly published and applicable to recognizable taxa.	interpretation of the ICNP, particularly in regard to status of names as validly published or otherwise.	
(b) **Organization.** The Judicial Commission (JC) shall consist of 12 commissioners elected by the members of the ICSP. No member of the JC may serve as a voting member of the EB-ICSP, and members of EB-ICSP may not serve on the JC.<br>(1) Commissioners are nominated by the members of the ICSP and elected to serve in three classes of four Commissioners, one class retiring upon the election of a new class. The terms of classes will begin on 1 September 2020 and every three years thereafter. Members of retiring classes are eligible for re-election.<br>(2) Immediately following the election of each new class of Commissioners, the Chair of the ICSP will organize the election of the officers of the JC from among its members. The officers will include the Chair, Vice-Chair and Secretary. The officers will serve until 1 September 2020 and every three years thereafter but may be reelected if their terms as commissioners have not expired.<br>(3) In the case of resignation or death of a Commissioner between elections, the	(b) **Organization.** The Judicial Commission (JC) shall consist of a standard 12 and a minimum of 9 commissioners elected by the members of the ICSP. No member of the JC may serve as a voting member of the ICSP, and voting members of ICSP may not serve on the JC. This restriction is not retroactive.<br>(1) Commissioners are elected by the members of the ICSP to serve in three classes of four Commissioners, one class retiring upon the election of a new class. A nomination for election requires the support of at least one Full Member of the ICSP and should summarize the relevant expertise of the candidate. The terms of classes begin on 1 September 2020 and every three years thereafter. Members of retiring classes are eligible for re-election.<br>(2) Immediately following the election of each new class of Commissioners, the Chair of the ICSP will organize the election of the officers of the JC from among its members. The officers will include the Chair, Vice-Chair and Secretary. The officers serve until 1 September 2020 and every three years thereafter but may be re-elected if their terms as commissioners have not expired.<br>(3) In the case of resignation, removal from office or death of a Commissioner between	This clarifies the minimum size of the JC in addition to the target size. It may not always be possible to find an immediate replacement for a resigning Commissioner. Twelve commissioners remains the target. The last addition is one of the pillars to implement the separation of powers been the legislature and the judiciary.<br><br>This addition clarifies the process for nominating a potential commissioner for election.

vacancy is filled by ballot of the members of the ICSP. A Commissioner of one class is eligible for election to a vacancy occurring in another class, but may only be a member of one class. Commissioners elected to fill a vacancy caused by resignation or death shall serve for the unexpired term of the vacancy.<br><br>(4) If any Commissioner cannot attend the meetings of the Judicial Commission, an Alternate having all the rights of a Commissioner except in the election of officers can be chosen.<br>(a) The Commissioner shall name an Alternate from the Full and Co-opted Members of the ICSP.<br>(b) If no name is submitted by the Commissioner, the JC may name an Alternate from the Full and Co-opted Members of the ICSP.<br>(c) No Alternate shall represent more than one absent Commissioner.	elections, the vacancy is filled by ballot of the members of the ICSP, although the JC shall continue to function if there are at least 9 Commissioners. A Commissioner of one class is eligible for election to a vacancy occurring in another class but may be a member of only one class. Commissioners elected to fill a vacancy caused by resignation or removal from office or death shall serve for the unexpired term of the vacancy.	This addition reflects the possibility of removal from office under Article 17 (formerly Article 18), and it clarifies the minimum size of the JC.<br><br>As above, alternates are not required now the JC is meeting regularly via electronic forums.
(c) **Duties of the Chair of the Judicial Commission**<br>(1) To preside at meetings of the JC.<br>(2) To prepare in collaboration with the Vice-Chair the agenda for meetings of the JC.	(c) **Duties of the Chair of the Judicial Commission**<br>(1) To preside at meetings of the JC.<br>(2) To prepare in collaboration with the Vice-Chair the agenda for meetings of the JC.	

(3) To participate in regular meetings of the EB-ICSP as a nonvoting member to communicate relevant matters regarding the JC and ICSP.<br><br>(4) To serve on the Editorial Board for the ICNP.<br><br>(5) To receive Requests for Opinions submitted to the IJSEM and, if applicable, to transmit these through the Secretary for Subcommittees on Taxonomy to appropriate Subcommittees on Taxonomy.<br>(6) To organize the JC's review of Requests for Opinions and Emendation of the ICNP as described below.<br><br>(7) To represent the JC on such International Committees, Boards or Commissions as may be organized to consider cooperation in biology in the solution of common problems of nomenclature and taxonomy, particularly to work with other similar Commissions or Executive Committees organized for action on problems on nomenclature in other biological sciences.<br>(8) To undertake such other duties as may from time to time be requested by the JC or the ICSP.	(3) To participate in regular meetings of the EB-ICSP as a non-voting member to communicate relevant matters regarding the JC and ICSP [see Article 4(d)].<br>(4) To serve on the Editorial Board for the ICNP [see Article 13].<br>(5) To receive Requests for Opinion accepted for publication by the IJSEM from its Editor-in-Chief, and to immediately transmit these to the JC for consideration.<br><br>(6) To organize the JC's consideration of Requests for Opinions [see Article 8] and recommendations regarding proposals for emendation of the ICNP [see Article 7(3)].<br>(7) To represent the JC on such international committees, boards or commissions as may be organized to consider cooperation in biology in the solution of common problems of nomenclature and taxonomy, particularly to work with other similar commissions or executive committees organized for action on problems on nomenclature in other biological sciences.<br>(8) To undertake such other duties as may from time to time be requested by the JC or the ICSP.	Changes intended to clarify the role of the chair and to adapt the wording to current practice. Some changes for clarifying the connection to other Articles.
(d) **Duties of the Vice-Chair of the Judicial Commission**<br>(1) To preside at meetings of the JC in the absence of the Chair. If both the Chair and the Vice-Chair are absent, the commissioners	(d) **Duties of the Vice-Chair of the Judicial Commission**<br>(1) To preside at meetings of the JC in the absence of the Chair. If both the Chair and the Vice-Chair are absent, the	

may elect an ad-hoc Chair to preside at the meeting.<br>(2) To assume all the duties of the Chair in the event of death or resignation of the Chair, until such time as a new Chair has been elected.<br>(3) To prepare in collaboration with the Chair and the Secretary of JC the agenda for meetings of the JC.<br>(4) To serve on the Editorial Board for the ICNP.<br>(5) To participate in regular meetings of the EB-ICSP as a nonvoting member to communicate relevant matters regarding the JC and ICSP.<br>(6) To undertake such other duties as may from time to time be requested by the JC or the ICSP.	commissioners may elect an ad hoc Chair to preside at the meeting.<br>(2) To assume all the duties of the Chair in the event of death or resignation of the Chair, until such time as a new Chair has been elected.<br>(3) To prepare in collaboration with the Chair and the Secretary of JC the agenda for meetings of the JC.<br>(4) To serve on the Editorial Board for the ICNP [see Article 13].<br>(5) To participate in regular meetings of the EB-ICSP as a non-voting member to communicate relevant matters regarding the JC and ICSP [see Article 4(d)].<br>(6) To undertake such other duties as may from time to time be requested by the JC or the ICSP.	Changes intended to clarify the connection to other Articles.
(e) **Duties of the Secretary of the Judicial Commission**<br>(1) To prepare and submit copies of the minutes of meetings of the JC for publication in the IJSEM.<br>(2) Co-ordinate voting by members of the JC and record the outcome of all votes.<br>(3) To participate in regular meetings of the EBICSP as a nonvoting member to communicate relevant matters regarding the JC and ICSP.	(e) **Duties of the Secretary of the Judicial Commission**<br>(1) To prepare and submit copies of the minutes of meetings of the JC for publication in the appropriate forum [see Article 4(d)].<br>(2) To co-ordinate voting by members of the JC and to record the outcome of all votes.<br>(3) To participate in regular meetings of the EB-ICSP as a non-voting member to communicate relevant matters regarding the JC and ICSP [see Article 4(d)].	Changes intended to clarify the connection to other Articles.

**Article 8**<br><br>**Requests for Opinions**<br><br>When directed by the ICNP to seek the consent of the JC or when necessary to resolve differences in opinion regarding the interpretation of the ICNP, the Opinion of the JC may be requested.<br><br>(a) The first step in the Request for Opinion is submission of a manuscript to the IJSEM requesting the opinion and providing the rationale.<br>(b) As deemed necessary, the Editor-in-Chief of the IJSEM will supervise the manuscript's review by members of the JC, the relevant Subcommittee of Taxonomy, and/or ad hoc reviewers. If the manuscript is recommended for publication, the Editor-in-Chief will ensure that the request is communicated to the Chair of the JC for consideration.	**Article 8**<br><br>**Requests for Opinions**<br><br>When necessary to resolve differences in opinion regarding the interpretation of the ICNP or, when necessary to clarify other questions regarding the nomenclature of prokaryotes, the Opinion of the JC may be requested.<br>(a) The first step in the Request for an Opinion is submission of a manuscript to the IJSEM requesting the opinion and providing the rationale.<br>(b) The Editor-in-Chief of the IJSEM will supervise the manuscript's review by members of the JC and, if deemed necessary, by members of the relevant Subcommittee on Taxonomy, and/or ad hoc reviewers. The manuscript should be rejected if it:<br>(1) fails to adequately present its case within the framework and appropriate application of the ICNP.<br>(2) revisits a previously denied Request for an Opinion without presenting significant new information on the case.<br>(3) requests the reconsideration of a previously issued Judicial Opinion but neither presents new evidence nor a new rationale.<br>If the manuscript is accepted for publication, the Editor-in-Chief will ensure that the request is communicated to the Chair of the JC for consideration immediately upon acceptance	Additional text clarifies the circumstances under which a Request for an Opinion may not be published in IJSEM

(c) If the manuscript is rejected upon review, seven members of the ICSP may request that the Request for Opinion proceeds further. In this case, the proposal is published in the IJSEM with a note identifying the members who support its publication.<br>(d) The proposal will then be reviewed by the JC within six months of publication. If approved by seven or more Commissioners, the proposal shall be submitted to the members of the ICSP for further consideration.<br>(e) If not approved by the JC, the reasons for rejection will be communicated to the ICSP and the authors of the Request for Opinion.<br>(f) The authors will then have three months to appeal the decision to the JC. If the JC now approves the Request for Opinion, it will be submitted to the ICSP along with the explanation for the initial rejection and the appeal.<br>(g) If the JC does not approve the Request for Opinion upon appeal, the authors may appeal directly to the ICSP within three months of the second rejection. In this case, they should communicate to the Executive Secretary the original Request for Opinion, the reasons for rejection by the JC, and the basis for appeal.<br>(h) The ICSP will make its decision within six months, which will be final. Notification of the decision shall be published in the IJSEM.	(i.e. in preprint or 'author accepted manuscript' form).<br>(c) If the manuscript is rejected upon review, seven voting Members of the ICSP may request that the Request for Opinion proceed further. In this case, the proposal is published in the IJSEM with a note identifying the Members who support its publication.<br>(d) The proposal will then be processed by the JC within six months of publication in the IJSEM. If approved by a majority of the Commissioners, the draft Judicial Opinion shall be submitted to the IJSEM for publication. JC ballots should not have abstention as an option.<br>(e) Each Judicial Opinion must describe the reasons for granting or denying the Request for an Opinion, or parts thereof. The rationale presented therein must be related to the pertinent clauses of the ICNP and, if applicable, to previous publications of the JC. Commissioners who disagree with the majority vote on the Judicial Opinion may include a minority report alongside the main Judicial Opinion.<br>(f) Decisions of the Judicial Commission may be overturned in a subsequent Judicial Opinion.	Clarification of the voting process. Removing abstentions is one of the measures to reduce the probability of a tied vote. Judges cannot abstain either.<br><br>As mentioned above, the need for Judicial Opinions to be approved by the voting members of the ICSP is questionable. In order to properly implement the separation of powers and to reduce the amount of bureaucracy, the processing of an RfaO is proposed here to be radically simplified.<br><br>Text in (e) – (g) in the older version is a rather cumbersome process in that it gives authors of RfaO two separate opportunities to contest the decision of the JC. Moreover, the Statutes have unfairly favored the authors of a RfaO, failing to consider that the interests of third parties may also be affected by a Judicial Opinion. The new version is much simpler, whilst still protecting

(i) In the absence of an opinion by the JC within one year of the publication of the Request for Opinion, the matter will transferred to the ICSP for a final decision. The JC will submit all correspondence on the matter to the Executive Secretary of the ICSP, who will arrange for the discussion and vote either by electronic means or at a plenary meeting within six months.	(g) In the absence of the submission of an Opinion for publication by the JC within half a year of the publication of the Request for an Opinion, the Executive Secretary of the ICSP shall reprimand the JC for the delay. In the absence of the submission of an Opinion for publication by the JC within one year of the publication of the Request for an Opinion, the Executive Secretary of the ICSP shall initiate an investigation by the EB-ICSP into the removal of members of the JC from office [see Article 17].	the 'right to reply' of the author(s) of RfaO and of any third party.<br><br>The new version also enforces much greater procedural and textual transparency in the Judicial Opinions themselves. It opens up the processing of Request for an Opinion to comments by third parties.
**Article 9**<br><br>**Publications Committee**<br><br>(a) The members of the Publications Committee shall be the Chair and Vice-Chair of the ICSP, the Chair and Vice-Chair of the JC, the Executive Secretary of the ICSP, the Secretary for Subcommittees on Taxonomy, and the Editor-in-Chiefs of the publications authorized by the ICSP.<br>(1) The Vice-Chair of the ICSP shall be Chair of the Publications Committee.<br>(2) The Executive Secretary shall be Secretary of the Publications Committee.<br>(3) Should a conflict of interest arise for any member of the Publications Committee, an alternate will be appointed from full	**Article 9**<br><br>**Publications Committee**<br><br>(a) The members of the Publications Committee shall be the Chair and Vice-Chair of the ICSP, the Chair and Vice-Chair of the JC, the Executive Secretary of the ICSP, the Secretary for Subcommittees on Taxonomy, and the Editor-in-Chiefs of the publications authorized by the ICSP. The Publisher of the IJSEM is entitled to an ex officio delegate to the Publications Committee.<br>(1) The Vice-Chair of the ICSP shall be Chair of the Publications Committee.<br>(2) The Executive Secretary shall be Secretary of the Publications Committee.<br>(3) Should a conflict of interest arise for any member of the Publications Committee, an alternate will be appointed from full	This addition reflects the terms of the current publishing agreement between ICSP and Microbiology Society (publisher of IJSEM).

members of the ICSP by the remaining members of the Committee.<br>(b) The functions of the Publications Committee shall be the following.<br>(1) Make recommendations to the ICSP for the establishment of such publications as deemed necessary for the advancement of the systematics of prokaryotes and their Editorial Boards.<br>(2) Be responsible for the publication of the *International Journal of Systematics and Evolutionary Microbiology* (IJSEM), *International Code of Nomenclature of Prokaryotes* (ICNP), the *Approved Lists of Bacterial Names* and the "Statutes of the International Committee on Systematics of Prokaryotes".<br>(3) Prepare an Annual Report and Financial Statement relating to publications for transmission to the Executive Secretary of the ICSP and the EB-ICSP.<br>(4) Submit a Report of the Publications Committee through the Executive Secretary of the ICSP for transmission to ICSP at each plenary meeting.	members of the ICSP by the remaining members of the Committee.<br>(b) The functions of the Publications Committee shall be the following.<br>(1) Make recommendations to the ICSP for the establishment of such publications as deemed necessary for the advancement of the systematics of prokaryotes and their Editorial Boards.<br>(2) Be responsible for the publication and dissemination of the *International Journal of Systematics and Evolutionary Microbiology* (IJSEM), *International Code of Nomenclature of Prokaryotes* (ICNP), the *Approved Lists of Bacterial Names* and the "Statutes of the International Committee on Systematics of Prokaryotes".<br>(3) To receive an annual report, including a financial statement from audited accounts, from the Publisher with regards to the publication of IJSEM and to report on this to the EB-ICSP.<br>(4) Submit a Report of the Publications Committee through the Executive Secretary of the ICSP for transmission to ICSP at each plenary meeting.	Change better reflects the reporting arrangements in the current publishing agreement between ICSP and Microbiology Society.
**Article 10**<br><br>The official publications of the ICSP are the IJSEM (formerly *International Journal of Systematic Bacteriology*), ICNP (formerly *International Code of*	**Article 10**<br><br>The official publications of the ICSP are the IJSEM (formerly *International Journal of Systematic Bacteriology*), ICNP (formerly *International Code of*	

Nomenclature of Bacteria), "Statutes of the International Committee on Systematics of Prokaryotes", and *Approved Lists of Bacterial Names*.	*Nomenclature of Bacteria*), "Statutes of the International Committee on Systematics of Prokaryotes", and *Approved Lists of Bacterial Names.*	
**Article 11**<br><br>**Sponsorship of Publications.** No publication of the ICSP or any of its organizations shall bear any indication of sponsorship by a commercial company, or institution connected in any way with a commercial company, except an acceptable acknowledgement of financial assistance. In cases of doubt, the EB-ICSP will make the final decision.	**Article 11**<br><br>**Sponsorship of Publications.** No publication of the ICSP or any of its organizations shall bear any indication of sponsorship by a commercial company, or institution connected in any way with a commercial company, except an acceptable acknowledgement of financial assistance. In cases of doubt, the EB-ICSP will make the final decision.	
**Article 12**<br><br>**Editorial Boards.** Editorial Boards shall be proposed by the EB-ICSP. Currently two Editorial Boards are recognized:<br>(a) The **Editorial Board for the *International Code of Nomenclature of Prokaryotes* (ICNP)**.<br>(b) The Editorial Board for the ***International Journal of Systematic and Evolutionary Microbiology* (IJSEM)**.	**Article 12**<br><br>**Editorial Boards.** Editorial Boards shall be proposed by the EB-ICSP. Currently two Editorial Boards are recognized:<br>(a) The Editorial Board for the ***International Code of Nomenclature of Prokaryotes* (ICNP).**<br>(b) The Editorial Board for the ***International Journal of Systematic and Evolutionary Microbiology* (IJSEM).**	
**Article 13**<br><br>Editorial Board for the *International Code of Nomenclature of Prokaryotes* (ICNP) shall consist of an Editor-in-Chief appointed by the EB-ICSP, the	**Article 13**<br><br>The Editorial Board for the *International Code of Nomenclature of Prokaryotes* (ICNP) shall consist of an Editor-in-Chief appointed by the EB-ICSP, the	

Chair and the Vice-Chair of the JC, and the Chair and Vice-Chair of the ICSP. The Editor-in-Chief shall be the Chair of the Editorial Board. The Board shall have power to co-opt.<br>(a) **Duties of Editor-in-Chief of the ICNP.** The Editor-in-Chief shall be responsible for the continuing revision of the ICNP, for its editing, and publication. The Editor-in-Chief shall submit the manuscript after approval by the Editorial Board for publication in book form and/or electronic form.<br>(1) To supervise consideration of proposals for emendation by the ICSP after publication in the IJSEM, including distribution of comments from the JC and members of the ICSP to the entire ICSP, and supervision of balloting among the ICSP.	Chair and the Vice-Chair of the JC, and the Chair and Vice-Chair of the ICSP. The Editor-in-Chief shall be the Chair of the Editorial Board. The Board shall have power to co-opt.<br>(a) **Duties of Editor-in-Chief of the ICNP.** The Editor-in-Chief shall be responsible for the continuing revision of the ICNP, for its editing, and publication. The Editor-in-Chief shall submit the manuscript after approval by the Editorial Board for publication in book form and/or electronic form.<br>(1) To supervise consideration of proposals for emendation by the ICSP after publication in the IJSEM, including distribution of comments from the JC and members of the ICSP to the entire ICSP, and supervision of balloting among the ICSP.	
(b) **Emendation of the ICNP.** The emendation of the Code shall follow this procedure.<br>(1) The first step in the emendation of the ICNP is submission of a manuscript to the IJSEM requesting emendation and providing the rationale.<br>(2) The Editor-in-Chief of the IJSEM will supervise the manuscript's review by the ICNP Editorial Board and/or ad hoc reviewers. If the manuscript is recommended for publication, the Editor of IJSEM will ensure that the request for emendation is communicated to the Editor-in-Chief of the ICNP, who will supervise its further consideration by the JC and ICSP.	(b) **Emendation of the ICNP.** The emendation of the Code shall follow this procedure.<br>(1) The first step in the emendation of the ICNP is submission of a manuscript to the IJSEM requesting emendation and providing the rationale.<br>(2) The Editor-in-Chief of the IJSEM will supervise the manuscript's review by the ICNP Editorial Board and/or ad hoc reviewers. If the manuscript is recommended for publication, the Editor-in-Chief of the IJSEM will ensure that the request for emendation is communicated to the Editor-in-Chief of the ICNP, who will supervise its further consideration by the ICSP and JC.	

(3) If the manuscript is rejected upon review, seven members of the ICSP may request that the proposal for emendation proceed further. In this case, the proposal is published in the IJSEM with a note identifying the members of the ICSP supporting it.	(3) If the manuscript is rejected upon review, seven members of the ICSP may request that the proposal for emendation proceed further. In this case, the proposal is published in the IJSEM with a note identifying the members of the ICSP supporting it.	
(4) The JC and members of the ICSP will have six months from the publication in the IJSEM to provide comments to the Editor-in-Chief of the ICNP. The authors of the proposal will then have two months to respond to the comments. After that time, the proposal and all comments and responses will be communicated to the members of the ICSP for further consideration.	(4) The members of the ICSP, the JC and other interested parties (including the authors of the proposal), will have three months from the publication in the IJSEM to provide comments to the Editor-in-Chief of the ICNP. The EB-ICSP will decide on the nature of any accompanying public discussion deemed appropriate. After that time, the proposal and all comments will be communicated to the members of the ICSP for further consideration.	Change better reflects current practice having an open discussion period (e.g., recent use of the Slack platform).
(5) The ICSP will make its decision within three months. If the proposal is not accepted, the authors may appeal the decision in writing within three months of notification of the decision of the ICSP.	(5) The ICSP will make its decision within three months. A summary of the debate and the outcome of the vote will be published in the IJSEM. Emendations take effect from the day of publication.	Addition highlights that changes are enacted by publication in IJSEM rather than compilation into a new edition of the ICNP.
	(6) If a proposal is not accepted, the authors or other parties may request the voting members of the ICSP to reconsider its decision by submitting a corresponding manuscript to the IJSEM [see Article 13(b)(1)]. This manuscript should present new evidence and/or a new rationale for supporting the emendation of the ICNP, and/or propose an adapted phrasing that addresses critical comments on the previous proposal(s).	Clarifies when an appeal regarding an ICSP decision is appropriate and the appropriate mechanism.

(6) The ICSP will have three months to consider the appeal, and its decision will be final.		
**Article 14**<br><br>**Editorial Board for *International Journal of Systematic and Evolutionary Microbiology*.** The Editorial Board shall consist of an Editor-in-Chief appointed by the EB-ICSP, the Vice-Chair of the ICSP, a representative appointed by the Publisher, and the Editors that are appointed as described below. The Editor-in-Chief will be Chair of the Editorial Board and may not serve concurrently as the Chair or Vice-Chair of the ICSP.<br>(a) The functions of the Editorial Board shall include implementation of the ICNP, including the General Considerations, Principles, Rules, Recommendations, and the appropriate aspects of the Appendices, within the IJSEM.<br>(b) The Editorial Board shall also implement recommendations made to it by the ICSP, JC or appropriate ad hoc committees formed by the ICSP to deal with specific matters relating to publications that appear in the journal.<br>(c) Nominations for Editorial Board should be sent to the Editor-in-Chief for consideration. The Editor-in-Chief appoints the Editors subject to the approval of the EB-ICSP.	**Article 14**<br><br>**Editorial Board for *International Journal of Systematic and Evolutionary Microbiology*.** The Editorial Board shall consist of an Editor-in-Chief appointed by the EB-ICSP, the Vice-Chair of the ICSP, a representative appointed by the Publisher, and the Editors that are appointed as described below. The Editor-in-Chief will be Chair of the Editorial Board and may not serve concurrently as the Chair or Vice-Chair of the ICSP.<br><br>(a) The functions of the Editorial Board shall include implementation of the ICNP, including the General Considerations, Principles, Rules, Recommendations, and the appropriate aspects of the Appendices, within the IJSEM.<br>(b) The Editorial Board shall also implement recommendations made to it by the ICSP, JC or appropriate Ad Hoc Subcommittees formed by the ICSP to deal with specific matters relating to publications that appear in the journal.<br>(c) Nominations for Editorial Board should be sent to the Editor-in-Chief for consideration. The Editor-in-Chief appoints the Editors subject to the approval of the EB-ICSP. If considered necessary, e.g. if an Editor does not perform her or his functions in a satisfactory way, the Editor-	Addition to clarify the role of Editor-In-Chief.

(d) The Editor-in-Chief shall be responsible for the review, acceptance and rejection of submitted manuscripts and for the supervision of the Editors.<br>(e) The Editor-in-Chief and the Editors shall be appointed for terms of up to five years subject to reappointment and earlier termination. Editors may serve for no more than two consecutive terms in any one capacity.<br>(f) Policy matters deemed important by the ICSP shall be communicated through the EB-ICSP to the Editor-in-Chief. Policy matters initiated by the Publisher shall be communicated through the Editor-in-Chief to the EB-ICSP.<br>(g) Editorial policy, whether initiated by the ICSP, the Publisher or elsewhere, shall be communicated through the Editor-in-Chief to the Editors.<br><br>(h) The Editor-in-Chief may serve on the Publisher's editorial policy committee representing the ICSP. The Editor-in-Chief shall act on behalf of the EB-ICSP, except in respect to the negotiation of contracts with the Publisher. In other transactions with the Publisher, the Editor-in-Chief shall forward copies of correspondence immediately to members of the EB-ICSP.<br>(i) The Publisher will submit all material for publication in the IJSEM to the Editor-in-Chief and Editors. The Editor-in-Chief and Editors shall have the power to refer such submissions to referees for assessment and reject such	in-Chief may remove an Editor subject to the approval of the EB-ICSP.<br>(d) The Editor-in-Chief shall be responsible for the review, acceptance and rejection of submitted manuscripts and for the supervision of the Editors.<br>(e) The Editor-in-Chief and the Editors shall be appointed for terms of up to five years subject to reappointment and earlier termination. Editors may serve for no more than two consecutive terms in any one capacity.<br>(f) Policy matters deemed important by the ICSP shall be communicated through the EB-ICSP to the Editor-in-Chief. Policy matters initiated by the Publisher shall be communicated through the Editor-in-Chief to the EB-ICSP.<br>(g) Editorial policy, whether initiated by the ICSP, the Publisher or elsewhere, shall be communicated through the Editor-in-Chief to the Editors.<br>(h) The Editor-in-Chief may serve on the Publisher's editorial policy committee representing the ICSP. The Editor-in-Chief shall act on behalf of the EB-ICSP, except in respect to the negotiation of contracts with the Publisher. In other transactions with the Publisher, the Editor-in-Chief shall forward copies of correspondence immediately to members of the EB-ICSP.<br>(i) The Publisher will submit all material for publication in the IJSEM to the Editor-in-Chief and Editors. The Editor-in-Chief and Editors shall have the power to refer such submissions to	

submissions that are deemed unsuitable for publication, provided that, in such instances, authors shall have the right of appeal via the Chair of the Editorial Board.<br>(j) The EB-ICSP shall be entitled to receive annually such part of the management accounts of the Publisher as relate to publication of the IJSEM and the audited accounts of the Publisher and to have access to such part of the books and records of the Publisher as are relevant to publication of the IJSEM at any time on giving reasonable notice and during normal business hours.<br><br>(k) Minutes of the Editorial Board meeting will be made available to the EB-ICSP.	referees for assessment and reject such submissions that are deemed unsuitable for publication, provided that, in such instances, authors shall have the right of appeal via the Chair of the Editorial Board.<br>(j) The EB-ICSP shall be entitled to receive annually such part of the management accounts of the Publisher as relate to publication of the IJSEM and the audited accounts of the Publisher and to have access to such part of the books and records of the Publisher as are relevant to publication of the IJSEM at any time on giving reasonable notice and during normal business hours.<br>(k) Minutes of the Editorial Board meeting will be made available to the EB-ICSP.	
**Article 16**<br><br>No one connected with a commercial firm may use his or her connection with the ICSP or any of its committees, commissions, and organizations, either as a member or officer, to advertise or promote his or her commercial interests in any way. Members of the EB-ICSP, JC, Editor-in-Chief and Editors of the IJSEM and ICNP shall submit a conflict of interest (COI) statement to the Chair of the ICSP upon the time of their appointment and must notify the Chair of any changes in their conflicts of interest during their terms of office.	**Article 15**<br><br>No one connected with a commercial firm may use his or her connection with the ICSP or any of its committees, commissions, and organizations, either as a member or officer, to advertise or promote his or her commercial interests in any way. Members of the EB-ICSP, JC, Editor-in-Chief and Editors of the IJSEM and ICNP shall submit a conflict of interest (COI) statement to the Chair of the ICSP upon the time of their appointment and must notify the Chair of any changes in their conflicts of interest during their terms of office.	Renumbered as it was noticed that there is no Article 15 in the current version of the statutes.

**Article 17**<br><br>**Amendments of the Statutes**<br><br>(a) Five or more members of the ICSP may initiate amendment of the Statutes by submission of a manuscript to the IJSEM requesting amendment and providing the rationale.<br>(b) As deemed necessary, the Editor-in-Chief of the IJSEM will supervise the manuscript's review by the EB-ICSP and/or ad hoc reviewers. Following the response of the authors to the reviewers' comments and submission of revisions if desired, the Editor-in-Chief will ensure timely publication in IJSEM and communication to the Executive Secretary of the ICSP for consideration by the full membership.<br><br>(c) The proposal will then be reviewed by the ICSP within six months of publication in the IJSEM, where at least 90 days is provided for deliberation by the members. Approval will require a vote of at least ten Members and shall be reached on the basis of a simple majority of the members voting.<br>(d) Notice of amendments approved by the ICSP shall be published in the IJSEM as a minute of a meeting of the ICSP or EB-ICSP.	**Article 16**<br><br>**Amendments of the Statutes**<br><br>(a) Five or more members of the ICSP may initiate amendment of the Statutes by submission of a manuscript to the IJSEM requesting amendment and providing the rationale.<br>(b) As deemed necessary, the Editor-in-Chief of the IJSEM will supervise the manuscript's review by members of the EB-ICSP and/or ad hoc reviewers. Following the response of the authors to the reviewers' comments and submission of revisions if desired, the Editor-in-Chief will ensure timely publication in IJSEM and communication to the Executive Secretary of the ICSP for consideration by the full membership.<br>(c) The proposal will then be reviewed by the ICSP within six months of publication in the IJSEM, where at least 45 days is provided for deliberation by the members. Approval will require a vote of at least ten Members and shall be reached on the basis of a relative majority of the members voting.<br>(d) Notice of amendments approved by the ICSP shall be published in the IJSEM as a minute of a meeting of the ICSP or EB-ICSP.	Renumbered<br><br>Clarification of the procedure. The entire EBICSP will hardly review such a manuscript.<br><br>Change intended to ease decision making.

**Article 18**<br><br>Officers of the ICSP, including members of the Executive Board, the Judicial Commission, and the Subcommittees of Taxonomy may be removed from office by a vote of the members of the ICSP. Reasons for removal from office include failure to perform the responsibilities of the office, using the office for purposes other than those specified in the Statutes, and violation of the conflict of interest statement.<br><br>(a) Removal from office is initiated by a letter from five or more members to the Executive Secretary or, if the Secretary is the subject of the complaint, to the Chair of the ICSP. The letter should specify the basis for removal. The officer will then have 30 days to respond. At the end of that time, the proposal to remove and the officer's response will be circulated to the members of the ICSP for a consideration. A vote of two-thirds of ten or more members is necessary to remove an officer.	**Article 17**<br><br>Officers of the ICSP, including members of the Executive Board, the Judicial Commission, the Subcommittees of Taxonomy and Ad Hoc Subcommittees may be removed from office by a vote of the Full Members of the ICSP. Reasons for removal from office include failure to perform the responsibilities of the office, using the office for purposes other than those specified in the Statutes, violation of the conflict-of-interest statement, and acting in any other way that significantly contravenes the Statutes.<br>(a) Removal from office is initiated by a letter from five or more Full Members to the Executive Secretary or, if the Secretary is the subject of the complaint, to the Chair of the ICSP. The letter should specify the basis for removal. The officer will then have 30 days to respond. At the end of that time, the proposal to remove and the officer's response will be circulated to the members of the ICSP for a consideration. A vote of two-thirds of ten or more members (i.e. a 'qualified majority' of those voting, where the threshold is two-thirds) is necessary to remove an officer.	Renumbered<br><br>Clarifications of the possible reasons of removal from office and of the kind of voting conducted.
